# Orthotolidin as a Blood Test

**Published:** 1913-11

**Authors:** 


### ABSTRACTS

Orthotolidin as a Blood Test. R. F. Ruttan and R. H. M. Hardesty, *Can. Med. Jour.*, November, 1912, recommend this reagent as a substitute for benzidin. Solutions are comparatively stable, keeping 3-4 weeks. If small quantities of hæmoglobin are present, the reaction is feeble, but increases gradually on standing. (Note. The real trouble with all blood tests is that they all depend on oxidation, with hydrogen peroxid in some form, under the influence of hæmoglobin acting as an oxidase. In stomach contents, fæces, etc., there is always the possibility of the presence of other oxidases than hæmoglobin, or, especially with guaiac, the color change may occur after some delay without the presence of hæmoglobin. Some authors go so far as to claim that a tube in which the hæmoglobin reaction has once occurred, will subsequently give the reaction, however carefully it is washed. Others insist that the tube must be perfectly dry when used, although water is obviously introduced in the test. It is interesting to note that Sir A. Conan Doyle makes one of his medical heroes discover a positive test for hæmoglobin. A good practical rule is to heat laboratory material in order to kill extraneous ferments, to cool, so as to prevent the hastening of the non-oxidase color change, and to pay no attention to a test that does not react within a minute and with reasonable positiveness. If this rule is followed, stomach contents, fæces and urine will usually give a negative blood test, a fact that reassures us as to the clinical reliability of a positive test which occurs without exception, so far as we have observed, if there is either macroscopic or microscopic evidence of blood; as well as faintly in cases in which blood is not thus readily detected. On the whole, we have found the phenolphthalin test most convenient, but it is a good plan to check it by the guaiac test.)

